# Correction to: Microbial function and genital inflammation in young South African women at high risk of HIV infection

**DOI:** 10.1186/s40168-022-01245-8

**Published:** 2022-03-09

**Authors:** Arghavan Alisoltani, Monalisa T. Manhanzva, Matthys Potgieter, Christina Balle, Liam Bell, Elizabeth Ross, Arash Iranzadeh, Michelle du Plessis, Nina Radzey, Zac McDonald, Bridget Calder, Imane Allali, Nicola Mulder, Smritee Dabee, Shaun Barnabas, Hoyam Gamieldien, Adam Godzik, Jonathan M. Blackburn, David L. Tabb, Linda-Gail Bekker, Heather B. Jaspan, Jo-Ann S. Passmore, Lindi Masson

**Affiliations:** 1 Division of Medical Virology, Department of Pathology, University of Cape Town, Cape Town, 7925 South Africa; 2 Division of Biomedical Sciences, University of California Riverside School of Medicine, Riverside, CA 92521 USA; 3 Computational Biology Division, Department of Integrative Biomedical Sciences, University of Cape Town, Cape Town, 7925 South Africa; 4 Division of Chemical and Systems Biology, Department of Integrative Biomedical Sciences, University of Cape Town, Cape Town, 7925 South Africa; 5 Division of Immunology, Department of Pathology, University of Cape Town, Cape Town, 7925 South Africa; 6 Centre for Proteomic and Genomic Research, Cape Town, 7925 South Africa; 7 Laboratory of Human Pathologies Biology, Department of Biology and Genomic Center of Human Pathologies, Mohammed V University, Rabat, Morocco; 8 Institute of Infectious Disease and Molecular Medicine (IDM), University of Cape Town, Cape Town, 7925 South Africa; 9 Centre for Infectious Diseases Research (CIDRI) in Africa Wellcome Trust Centre, University of Cape Town, Cape Town, 7925 South Africa; 10 Seattle Children’s Research Institute, University of Washington, Seattle, WA 98101 USA; 11 Bioinformatics Unit, South African Tuberculosis Bioinformatics Initiative, Stellenbosch University, Stellenbosch, 7602 South Africa; 12 DST–NRF Centre of Excellence for Biomedical Tuberculosis Research, Stellenbosch University, Stellenbosch, 7602 South Africa; 13 Desmond Tutu HIV Centre, University of Cape Town, Cape Town, 7925 South Africa; 14 Centre for the AIDS Programme of Research in South Africa, Durban, 4013 South Africa; 15 National Health Laboratory Service, Cape Town, 7925 South Africa; 16 Disease Elimination Program, Life Sciences Discipline, Burnet Institute, 85 Commercial Road, Melbourne, Victoria 3004 Australia; 17 Central Clinical School, Monash University, Melbourne, 3004 Australia

**Correction to: Microbiome 8, 165 (2020)**

**https://doi.org/10.1186/s40168-020-00932-8**

The author reported that Additional file 2 for this article [1] is a draft copy of Additional file 1. The correct additional file 2 should have been an excel file. The original article has been updated.

## Supplementary Information

**Additional file 2.**
